# Case report: Traumatic median neuropathy in the distal forearm after massage therapy

**DOI:** 10.3389/fneur.2022.959919

**Published:** 2022-09-21

**Authors:** Mi Rim Suh, Jun Hyeong Song, Yongbum Park, Seok Hyeon Lee, Jaeki Ahn

**Affiliations:** Department of Rehabilitation Medicine, Sanggye Paik Hospital, Inje University College of Medicine, Seoul, South Korea

**Keywords:** median neuropathy, mononeuropathy, massage, ultrasound, nerve conduction study, magnetic resonance imaging

## Abstract

Median nerve damage caused by trauma is rare, especially after the massage therapy. There have been no reports of median neuropathy in the distal forearm following massage therapy. A 61-year-old man developed paresthesia and numbness in the right hand after two sessions of massage therapy. Electrophysiologic studies, ultrasound, and magnetic resonance imaging were used to localize and confirm the median nerve lesion in the distal forearm. Ultrasound-guided perineural steroid injection and oral pregabalin were administered. At the 1-month follow-up, more than 80% of the sensory symptoms had resolved, and the results of the sensory nerve conduction study and ultrasound showed improvement. Although massage-induced mononeuropathy is uncommon, massage therapy should be performed carefully to avoid complications.

## Introduction

Mononeuropathy of the peripheral nerves is usually caused by local compression, inflammation, trauma, tumors, or other etiologies (1, 2). Compressive neuropathies tend to occur at specific locations where the nerve is likely to be trapped when passing through narrow areas, such as the fibro-osseous or fibromuscular tunnel (3). On the contrary, if the nerve is superficial or has a long course, it can easily be damaged by strong external forces such as fractures or other trauma (4).

In addition, neuropathy may occur when a small external force is applied over a long time. Some cases of neuropathy occurring after undergoing massage for treating musculoskeletal pain have also been reported (5–11). However, to our knowledge, no cases of injury in the main trunk of the median nerve following the massage have been reported in the past. Here, we report the first case of median neuropathy in the distal forearm following massage therapy.

## Case description

A 61-year-old man presented with numbness and paresthesia in his right hand after the massage therapy. A month ago, he underwent massage therapy for his distal forearm to relieve the pain in his right finger. The masseur pressed the palmar side of the right distal forearm strongly using the elbow, and the massage session lasted for 90–120 min. During the massage therapy, sensory disturbance developed in the right hand. The patient complained to the masseur after the massage therapy; however, the masseur said it would improve over time. After 1 week, another massage session was performed for the same area. Afterward, the symptoms worsened, and the patient visited our clinic.

On physical examination, the right distal forearm, where the patient received the massage, was slightly swollen. There was no focal atrophy in the muscles, including the thenar muscles. Hypoesthesia and paresthesia were assessed in the palmar aspect of the right hand and the first three and one-half digits. Except for sensory symptoms in the hand, there were no abnormalities in the patient's motor function and reflexes. There were no other neurologic deficits, such as cranial nerve dysfunction and bladder or bowel symptoms. His medication history included the use of beta-blockers, aspirin, and statins for hypertension and dyslipidemia. Blood tests including complete blood count, blood urea nitrogen, electrolytes, and metabolic profile, showed no abnormalities.

**Figure 1:**
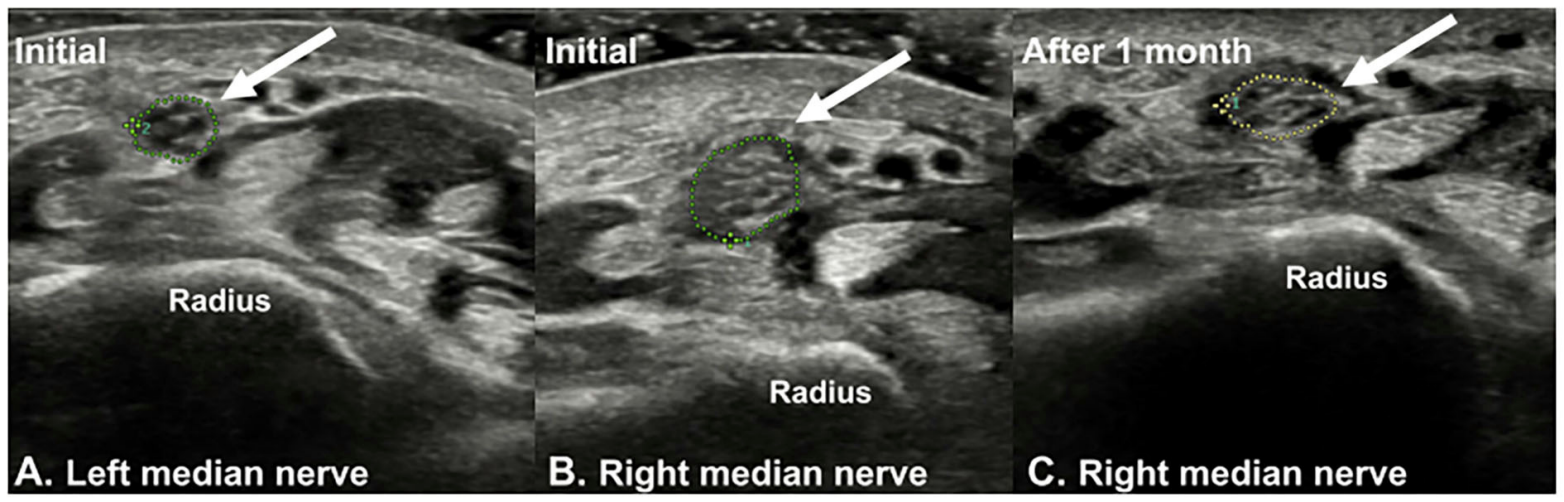
Ultrasound imaging of **(A)** left median nerve and **(B)** right median nerve (arrow). The sonographic findings showed a hypoechoic swelling of the right median nerve at the distal forearm level (4 cm above the distal wrist crease). At this level, the cross-sectional area (CSA) of the right median nerve was 0.19 cm^2^, and that of the left side was 0.09 cm^2^. **(C)** At the 1-month follow-up, the CSA of the right median nerve was 0.12 cm^2^.

**Figure 2:**
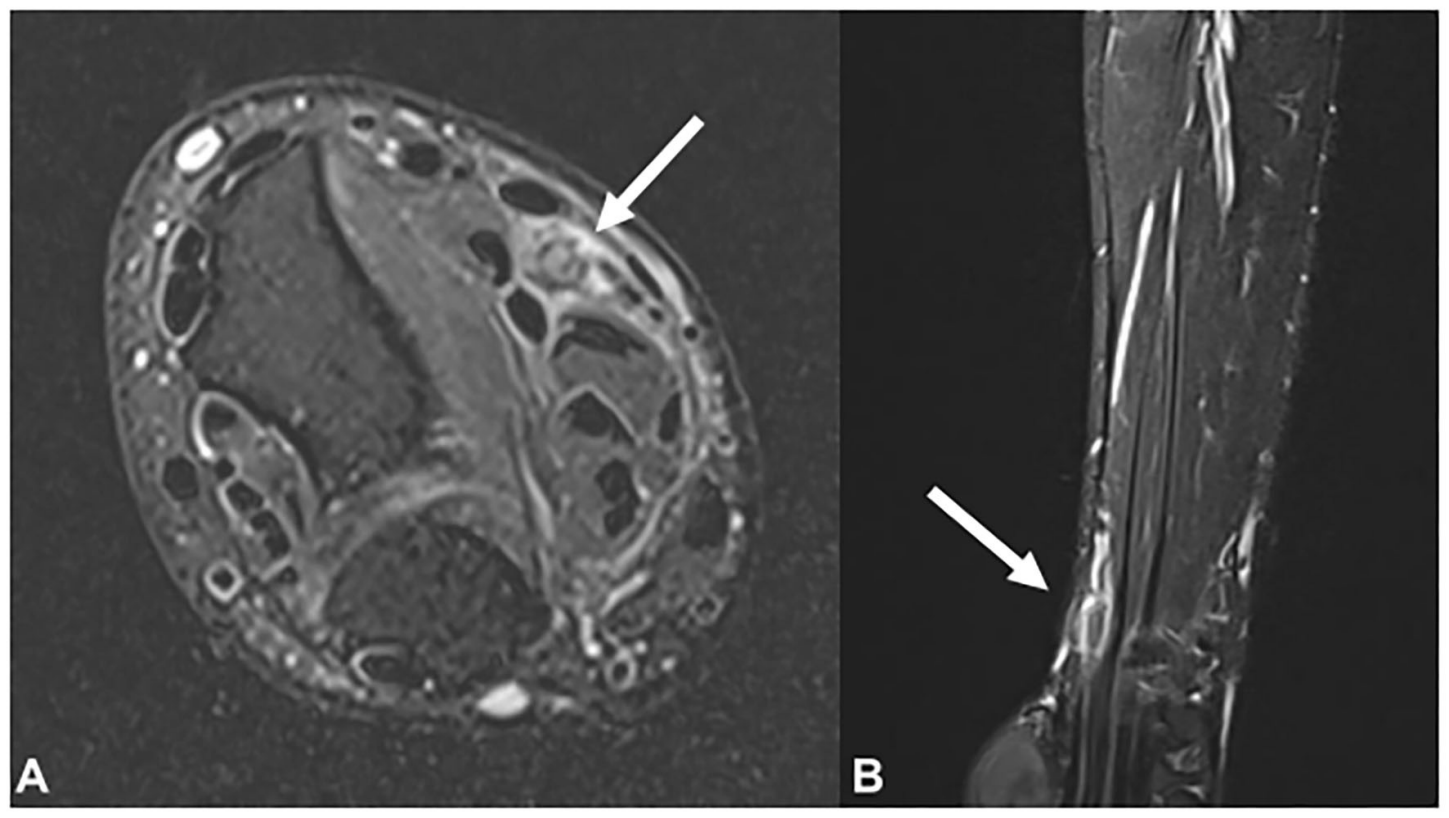
Magnetic resonance imaging findings of the right median nerve (arrow) in the **(A)** T2-weighted axial view and **(B)** T2-weighted sagittal view. Focal thickening and signal change in the right median nerve is observed at the distal radius level.

Plain radiographs showed no signs of fracture or other abnormalities. Ultrasound examination revealed a hypoechoic, swollen right median nerve, 4 cm proximal to the wrist crease. The cross-sectional area (CSA) of the right median nerve at the lesion site was 0.19 cm^2^, while that of the left median nerve was 0.09 cm^2^ (Figure 1). Magnetic resonance imaging (MRI) of the right forearm revealed edema in the surrounding fat tissue and a focal defect in the epineurium of the right median nerve, at the distal radius level (Figure 2).

Furthermore, nerve conduction study (NCS) and electromyography (EMG) were performed 1 month after the onset of symptoms. Antidromic NCS of the median nerve was performed for the second finger by stimulating it from the forearm. To identify changes in the results with regard to the lesion site, two stimulation sites were designated: one was 14 cm above the recording electrode (the distal aspect of the lesion) and the other was 18 cm above the recording electrode (the proximal aspect of the lesion). In the right median sensory study, the onset latency and amplitude were 2.60 ms and 49.0 μV, respectively, when stimulated from 14 cm above, and 3.65 ms and 15.6 μV, respectively, when stimulated from 18 cm above (Table 1). In the left median sensory study, the onset latency and amplitude were 2.50 ms and 51.4 μV, respectively, when stimulated from 14 cm above, and 3.20 ms and 46.0 μV, respectively, when stimulated from 18 cm above. Compared to the left median NCS, the right median NCS showed delayed onset latency and lower amplitude. However, in the motor NCS, the median nerves of both sides exhibited normal onset latency, amplitude, and nerve conduction velocity. On EMG, the muscles of the right upper limb, including the abductor pollicis brevis, showed normal action potentials and interference patterns without abnormal spontaneous activity (Table 2). No abnormal denervation pattern was observed in the cervical paraspinal muscles.

**Table 1:** Nerve conduction study of upper extremities.

**Sensory NCS**				**Motor NCS**			
**Nerve**	**Onset latency (ms)**	Distance* **(cm)**	**Amplitude (**μ**V)**	**Nerve** **(Record site)**	**Onset latency (ms)**	**NCV** **(m/s)**	**Amplitude (mV) (Distal/Proximal)**
**At initial**							
Rt. median nerve	2.60	14.0	49.0	Rt. median nerve (APB)	3.0	54.1	14.6/10.7
	3.65	18.0	15.6				
Lt. median nerve	2.50	14.0	51.4	Lt. median nerve (APB)	2.9	55.6	15.2/11.7
	3.20	18.0	46.0				
Rt. ulnar nerve	2.30	14.0	50.4	Rt. ulnar nerve (ADM)	2.6	63.8	15.2/12.6
Rt. radial nerve	1.80	14.0	31.5	Rt. radial nerve (EIP)	1.9	69.2	5.0/4.8
**At 1 month follow up**							
Rt. median nerve	2.50	14.0	49.5	Rt. median nerve (APB)	3.1	57.1	14.9/11.0
	3.40	18.0	24.8				
Lt. median nerve	2.40	14.0	53.5	Lt. median nerve (APB)	2.9	55.6	15.4/11.9
	3.20	18.0	47.0				

*Distance between stimulation site and active electrode.

NCS, nerve conduction study; NCV, nerve conduction velocity; Rt, right; APB, abductor pollicis brevis; Lt, left; ADM, abductor digiti minimi; EIP, extensor indicis proprius.

**Table 2:** Findings of needle electromyography in right upper extremity.

**Muscle**	**IA**	**ASA**	**MUAP**	**Recruitment**
Abductor pollicis brevis	Normal	–	Normal	Normal
Abductor digit minimi	Normal	–	Normal	Normal
Pronator quaratus	Normal	–	Normal	Normal
Flexor carpi radialis	Normal	–	Normal	Normal
Extensor carpi radialis	Normal	–	Normal	Normal
Biceps brachii	Normal	–	Normal	Normal
Triceps brachii	Normal	–	Normal	Normal

IA, insertion activity; ASA, abnormal spontaneous activity; MUAP, motor unit action potential.

The patient was diagnosed with right median neuropathy of the distal forearm based on the radiologic and electrophysiologic examinations. Pregabalin (100 mg) thrice a day was prescribed for the paresthesia of the right hand. The patient's symptoms continued, so an ultrasound-guided perineural injection was performed 2 weeks later. The affected distal forearm was sterilized with chlorhexidine 2%. Then, using the transverse approach, we identified the maximal swelling point of the median nerve. A 26-gauge needle was inserted *via* radial approach, and a mixture containing 2 cc of 0.5% lidocaine and 2.5 mg of dexamethasone was injected.

At the 1-month follow-up, the patient showed more than 80% improvement in the symptoms, and follow-up sensory NCS revealed improvement in the onset latency and amplitude of the median nerve (Table 1). On sonographic examination, the CSA of the right median nerve was 0.12 cm^2^, indicating improvement compared to the initial presentation (Figure 1). Further examinations could not be performed as the patient was lost to follow-up.

## Discussion

This is the first case report of median neuropathy in the distal forearm caused by massage therapy, which is a rare cause of neuropathy. Our patient developed paresthesia and hypoesthesia in his right hand immediately after one session of massage therapy. The masseur strongly pressed the palmar side of the right distal forearm, where the median nerve is located. The patient's sensory disturbances were consistent with the symptoms of median neuropathy. His occupation was an office worker, and he was right-handed. There were no predisposing factors related to the neuropathy; he had never felt sensory disturbances in his hand before massage therapy and had no previous history of trauma to his forearm. And the patient had never been diagnosed with carpal tunnel syndrome or cervical radiculopathy, and the existing finger pain was caused by the trigger finger. Electrophysiologic studies, ultrasound, and MRI were used to localize the site of the median nerve injury and exclude other etiologies, such as space-occupying lesions. Moreover, the prognosis of neuropathy was determined through electrophysiologic examinations, including NCS and EMG.

Massage is defined as a systemic form of touch or manipulation applied to the soft tissue. Massage therapy promotes local circulation in tissues and relieves muscle spasms and soft-tissue adhesions. Mild complications associated with massage therapy, namely focal discomfort or pain, fatigue, headache, dizziness, and nausea, have been reported in the literature (12). Moreover, several cases have reported severe complications of massage, namely, spinal cord injury, hepatic hematoma, and embolism of the retinal and cerebral arteries (12–15).

Some cases have reported mononeuropathy as a complication of massage therapy, but no case of traumatic injury to the main trunk of the median nerve has been reported worldwide (6–11). In previously reported cases, the duration of the massage ranged from 5 min to 1 h. The lesion locations also varied, namely those of the radial nerve, posterior interosseous nerve, spinal accessory nerve, and sciatic nerve. There has been only one case report on injury to the recurrent motor branch of the median nerve due to massage (9). In some cases of a massage-related mononeuropathy, symptoms of focal weakness, abnormalities in the motor NCS, and denervation potentials on needle EMG indicated axonotmesis of the nerve (6–11).

In our case, median neuropathy developed after direct massage of the distal forearm for 90–120 min. The patient showed delayed latency and low amplitude on sensory NCS without any abnormalities on motor NCS and needle EMG, and the degree of nerve damage, in this case, was indicative of neurapraxia according to the Seddon's classification (16). When stimulating the distal part of the lesion, amplitude and latency was normal in the right median sensory study, because there was no axonal loss and Wallerian degeneration. Neurapraxia is the mildest type of peripheral nerve injury induced by focal demyelination or ischemia. The course after neurapraxia is usually favorable, and the majority of the recovery occurs within 2–3-months (17). In this case, our patient experienced more than 80% improvement in the symptoms during the 1-month follow-up. In addition, the sensory NCS and ultrasound results showed improvement.

The patient underwent conservative treatment with medications and ultrasound-guided perineural corticosteroid injection. Peripheral nerve injury results in the inflammatory/immune reaction at the lesion of neural tissue that can induce a secondary injury to inhibit the regeneration of the nerve (18). Therefore, suppression of the immune response is critical for the recovery of nerve damage. Dexamethasone is an anti-inflammatory agent, and several studies have reported that this drug promotes neural repair and reduces edema in nerve lesions (18, 19). Since the patient's symptom improvement with the medication was insignificant, the perineural injection of dexamethasone was performed.

Massage is a part of alternative medicine and is used as a treatment to control musculoskeletal pain in many countries, but only a few complications of massage have been reported to date. In particular, minor injuries such as neurapraxia, in this case, axonotmesis, and more serious nerve damage may occur; hence, massage therapists should be well aware of the anatomy of the nerves, vessels, and muscles and perform massage therapy carefully in those areas. If any neurologic signs, namely, pain, motor weakness, or even mild sensory symptoms, occur during massage therapy, it should be discontinued immediately. We have reported a rare case of median neuropathy in the distal forearm that occurred at an unusual location after massage treatment with a review of the literature.

## Data availability statement

The original contributions presented in the study are included in the article/supplementary material, further inquiries can be directed to the corresponding author.

## Ethics statement

Written informed consent was obtained from the individual(s) for the publication of any potentially identifiable images or data included in this article.

## Author contributions

MS, JS, YP, and JA: conceptualization. YP and JA: supervision. MS, JS, and SL: visualization and writing—original draft. MS, YP, and JA: writing—review and editing. All authors contributed to the article and approved the submitted version.

## Conflict of interest

The authors declare that the research was conducted in the absence of any commercial or financial relationships that could be construed as a potential conflict of interest.

## Publisher's note

All claims expressed in this article are solely those of the authors and do not necessarily represent those of their affiliated organizations, or those of the publisher, the editors and the reviewers. Any product that may be evaluated in this article, or claim that may be made by its manufacturer, is not guaranteed or endorsed by the publisher.
